# Efficient *Agrobacterium*-Mediated Methods for Transient and Stable Transformation in Common and Tartary Buckwheat

**DOI:** 10.3390/ijms26094425

**Published:** 2025-05-06

**Authors:** Sara Leite Dias, Paride Rizzo, John Charles D’Auria, Andriy Kochevenko

**Affiliations:** Leibniz Institute of Plant Genetics and Crop Plant Research (IPK) Gatersleben, 06466 Seeland, Germany; leite@ipk-gatersleben.de (S.L.D.); rizzo@ipk-gatersleben.de (P.R.)

**Keywords:** buckwheat, *Fagopyrum esculentum*, *Fagopyrum tataricum*, transient gene expression, genetic transformation, *Agrobacterium*, metabolic engineering, gramine production

## Abstract

Buckwheat is a promising crop with grains that are rich in nutrients and bioactive compounds. Genome sequence data for common and Tartary buckwheat have recently become available. Currently, there is a critical need for the development of a simple and reliable transient gene expression protocol, as well as a stable genetic transformation method, to facilitate metabolic engineering of bioactive compounds, functional analysis of genes, targeted editing, and, in a long-term perspective, to accelerate the breeding process in buckwheat. In this paper, we report optimized methods for *Agrobacterium*-mediated transient and stable transformation of *Fagopyrum esculentum* and *F*. *tartaricum*. Leaf and cotyledon tissues were infiltrated with an *A*. *tumefaciens*-bearing construct containing *eGFP* and *GUS* reporter genes. Histochemical staining and Western blotting were used to confirm the expression of reporter proteins. We also demonstrate the usefulness of the developed method for engineering the gramine biosynthetic pathway in buckwheat. *HvAMIS* and *HvNMT* genes were transiently expressed in buckwheat leaves, and the de novo production of gramine was confirmed by LC-MS. Moreover, in planta genetic transformation of common and Tartary buckwheat with a reporter gene (*eGFP*) and selectable marker gene (*NptII*) was achieved by *Agrobacterium*-mediated vacuum infiltration. Genomic integration of the construct was confirmed by polymerase chain reaction (PCR), whereas the production of eGFP was confirmed by fluorescence microscopy.

## 1. Introduction

Buckwheat is classified as a pseudocereal within the *Polygonaceae* family. The genus *Fagopyrum* encompasses two cultivated species of buckwheat: common buckwheat (*Fagopyrum esculentum* Moench) and Tartary buckwheat (*Fagopyrum tataricum* (L.) Gaertn.) [1]. Buckwheat seeds are notable for their high protein content, which includes essential amino acids, such as lysine, leucine, and arginine. They also contain vitamins like vitamin E, thiamine, and riboflavin, various minerals, and a broad range of phenolic compounds including rutin, epicatechin, quercitrin, quercetin, fagopyrin, and phenolic acids [2,3]. Polyphenolic compounds found in buckwheat have been reported to exhibit anti-inflammatory, antioxidant, antiviral, antibacterial, and cardiovascular health-promoting activities [4,5]. Buckwheat is a valuable source of functional food products and bioactive compounds.

The development of stable and transient transformation techniques is of profound importance in plant biotechnology, particularly for enhancing the production of bioactive compounds through metabolic engineering [6]. These methods empower researchers to manipulate biosynthetic pathways, enabling the production of valuable bioactive compounds in a sustainable and scalable manner. Given buckwheat’s unique composition of specialized metabolites, such advancements position it as an ideal candidate for both functional studies and biotechnological applications.

In recent decades, significant advancements have been made in the development of buckwheat genetic and genomic resources. These resources include various mutant collections [7,8,9], several linkage maps constructed using multiple molecular markers [10], and reference genomes of *F*. *esculentum* [8,11], *F*. *esculentum* var. homotropicum [12], and *F*. *tataricum* [13]. These data sources offer researchers opportunities to explore gene function and regulatory mechanisms. Moreover, combining traditional and genomics-assisted breeding should facilitate the development of new buckwheat varieties with improved yield and quality [14].

Genetic engineering of plants is an effective tool for understanding gene function, plant breeding, and molecular farming [15,16]. Although virus-based vector systems have been successfully applied in plants [17], transgenesis, cisgenesis/intragenesis, and genome editing (GE) primarily rely on conventional binary vectors and *Agrobacterium*-mediated transformation [18,19,20].

*Agrobacterium*-mediated transient transformation is a straightforward and rapid method for analyzing gene function and producing recombinant proteins [21,22]. This technique is particularly useful for plant species for which developing in vitro regeneration and transformation protocols remains challenging. Protoplast-based transient expression systems are commonly used for buckwheat. Transient gene expression in *F*. *esculentum* protoplasts has been effectively employed to analyze the subcellular localization of transporters involved in aluminum tolerance [23,24] and the transcriptional activation ability of the MYBF1 transcription factor [25]. Although protoplast-based transient expression is very useful for the functional gene analysis, it is not the optimal platform for the production of bioactive compounds through metabolic engineering.

Stable in vitro genetic transformation of a limited number of buckwheat genotypes using *Agrobacterium* cells has been reported by various authors. Several *A*. *rhizogenes* strains have been successfully used to generate transgenic hairy root cultures of buckwheat. Such root cultures have been widely used to produce specialized metabolites and investigate their regulation [26,27,28,29,30]. Additionally, efficient in vitro transformation protocols with *A*. *tumefaciens* have been developed for CRISPR/Cas9-mediated gene editing in *F*. *tataricum* [31] and expression of transgenes in *F*. *esculentum* [32,33,34]. However, it should be noted that often this technique is based on a regeneration protocol specific to a particular cultivar and may not be applicable to other varieties. In addition, the in vitro transformation technique is labor-intensive, time-consuming, requires special skills, and incurs high costs.

In contrast to conventional in vitro transformation, the in planta (also known as in situ) transformation method is technically simple, quick, and cost-effective [35]. Although in planta transformation avoids potential tissue culture-associated disadvantages such as plant regeneration and somaclonal variation, this method is characterized by a relatively low transformation rate [36]. Germline transformation leads to the generation of hemizygous T1 transgenic seedlings [37]. It is believed to be genotype-independent [35,36]; therefore, superior plant phenotypes expressing transgenes or edited genes can be produced for any accession, and subsequently such lines can be directly involved in crop improvement programs. Unfortunately, generating transgenic plants in buckwheat via the in planta transformation approach is still not a routine practice. To date, only a few studies have reported successful in planta transformations in *F*. *esculentum*. Bratić et al. [38] reported transient transformation of common buckwheat through *Agrobacterium*-mediated infiltration using a β-glucuronidase-expressing vector. The first stable *F*. *esculentum* transformants were produced by piercing the shoot apical meristem with a needle and subsequently inoculating with *A*. *tumefaciens* carrying a vector containing the β-glucuronidase reporter gene and neomycin phosphotransferase II (*NPTII*) gene for plant selection [39]. Later, the same genotype, *F*. *esculentum* var. Shinano No. 1, was successfully transformed with the rice MADS-box gene to analyze its potential role in flower development [40]. Recently, Zhou [41] reported a transformation of common buckwheat using the *Agrobacterium*-mediated floral dip method. The generated transgenic plants exhibited GFP fluorescence and resistance to the herbicide, Basta. In planta transient transformation of Tartary buckwheat was also reported. Yao et al. [42] demonstrated that transient expression of *FtMAPK1* significantly increased the antioxidant enzyme activity in Tartary buckwheat leaves under abiotic stress. Moreover, Wu et al. [43] described transient transformation of *F*. *tartaricum* cotyledons and seeds with variety of constructs containing *eYFP*/*mKate2* or *GUS* reporter genes, respectively.

A more popular system for transient expression is *Nicotiana benthamiana*, which is the most widely used model for gene expression studies in plants because of its numerous advantages. It is highly susceptible to *A*. *tumefaciens*, which facilitates efficient gene transfer and rapid expression of introduced genes. This makes the high-throughput screening of gene functions and protein interactions ideal. Additionally, *N*. *benthamiana* has a relatively short life cycle and is easy to grow under controlled conditions, allowing for rapid experimental turnaround. Its large leaves provide ample tissue for infiltration and analysis, making it suitable for various molecular biology techniques such as protein localization studies and virus-induced gene silencing (VIGS) [44]. However, there are some disadvantages in using *N*. *benthamiana*. Its responses to gene expression and pathogen infection may not always be representative of those of other plant species, limiting the applicability of the findings [45]. Moreover, its high susceptibility to pathogens can sometimes complicate experiments by introducing unintended variables [46]. Lastly, manipulating specialized metabolic pathways in *N*. *benthamiana* may be complicated by the endogenous pathways for pyridine alkaloids like nicotine and anabasine [47].

Common and Tartary buckwheat can be an alternative model to *N*. *benthamiana* as an ortholog system for transient and stable transformation. This system can be used in a wide range of applications, including functional validation of genes in plants. The absence of pyridine alkaloids in buckwheat might facilitate the isolation of new compounds and proteins from transgenic plants, avoiding complications associated with the secondary metabolites of *N*. *benthamiana*. Therefore, we conducted a study to optimize strategies for in planta *Agrobacterium*-mediated transient and stable transformations of both *F*. *esculentum* and *F*. *tataricum*. In this study, we applied this method for the genetic transformation of buckwheat with a reporter gene (*eGFP*) and a selectable marker gene (*NptII*) via *Agrobacterium*-mediated vacuum infiltration. The protocol for transient transformation was tested by infiltrating leaves and cotyledons with *A*. *tumefaciens* carrying a construct containing *eGFP* and *GUS* reporter genes. We used the developed protocol to engineer gramine biosynthesis in buckwheat. We transiently co-expressed the *HvAMIS* and *HvNMT* genes, which resulted in gramine production in buckwheat leaves. By developing this simple and reliable technique, we aim to ease high-throughput genetic manipulation of buckwheat. We anticipate that transformation protocols will be highly valuable for the functional analysis of genes and manipulation of metabolic pathways in plants. This can further support buckwheat breeding efforts to enhance agronomic traits, including the production of compounds beneficial for human health.

## 2. Results

### 2.1. Transient Expression of Reporter Genes

**Figure 1:**
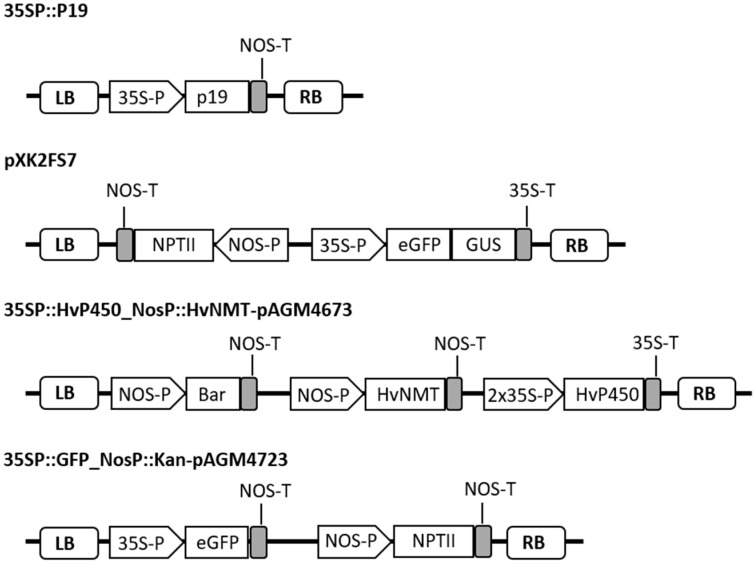
Schematic representation of the T-DNA region of the transformation vectors used in this study. “::” indicates a genetic fusion, either between a promoter and a gene, or between genes. LB and RB correspond to the left and right borders of the T DNA; 35S-P and 35S-T, cauliflower mosaic virus 35S promoter and terminator, respectively; NOS-P and NOS-T, nopaline synthase promoter and terminator, respectively; p19, tomato bushy stunt virus p19 silencing suppressor; Bar, phosphinothricin *N*-acetyltransferase gene; *eGFP*, enhanced green fluorescent protein gene; *HvNMT*, *N*-methyltransferase gene; and *HvP450*, aminomethylindole synthase gene (*HvAMIS*).

**Figure 2:**
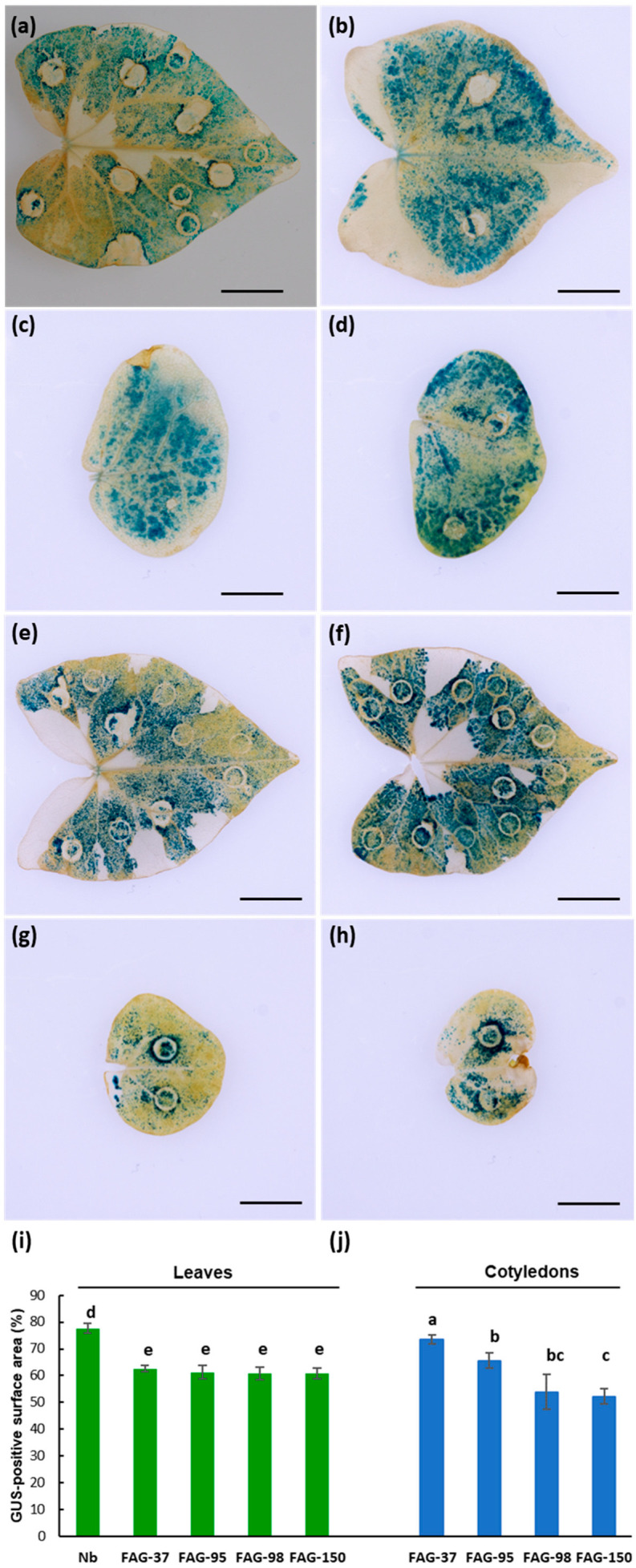
Transient GUS expression assay of *Agrobacterium*-infiltrated buckwheat cotyledons (in blue) and leaves (in green). (**a**–**h**) Histochemical localization of transient GUS activity in infiltrated *F*. *esculentum* FAG-37, FAG-95 (**a**–**d**) and *F*. *tataricum* FAG-98, FAG-150 (**e**–**h**) samples, respectively. The scale bars correspond to 10 mm. (**i**,**j**) The effect of genotype on percent area of GUS-positive foci. The samples were syringe-infiltrated with 0.6 OD600 of GV2260 harboring pXK2FS7. Data are means of three biological replicates and error bars represent SE. Different letters indicate significant differences (*t*-test, *p* < 0.05).

Cotyledons and leaves of various buckwheat accessions (FAG-37, FAG-95, FAG-98, and FAG-150) were transiently transformed using the pXK2FS7 GFP-GUS vector (Figure 1). Histochemical staining revealed large and numerous blue-stained areas in all infiltrated samples of both common and Tartary buckwheat (Figure 2a–h). In cotyledons, the percentage of GUS-positive surface area was higher in common buckwheat accessions (12–21%) than in Tartary accessions. Accession FAG-150 had the lowest percentage of stained areas among the genotypes (52.2%), whereas FAG-37 showed a significantly higher transgene expression in transiently transformed cotyledons (73.4%) (Figure 2j). In contrast, the percentage of GUS-positive leaf surface area was similar across all tested accessions, ranging from 60.9% to 62.6% (Figure 2i).

To prove accumulation of GFP protein, the total proteins from the infiltrated leaf tissues were extracted and analyzed by Western blotting. Probing the blot membranes with a GFP-specific antibody detected the eGFP-GUS fusion protein at the predicted molecular mass of 95.35 kDa (Figure S1). These results, along with the histochemical staining data, confirmed the synthesis of functional recombinant eGFP-GUS protein in the infiltrated buckwheat tissues.

### 2.2. Assembly of the Gramine Biosynthesis Pathway

**Figure 3:**
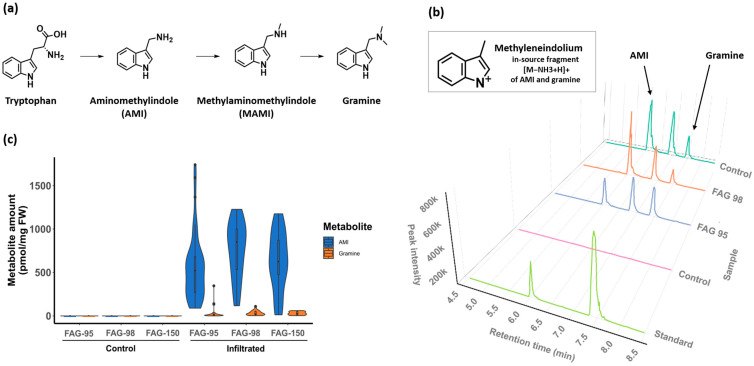
Transient expression of *HvAMIS* and *HvNMT* leads to the biosynthesis of gramine in buckwheat. (**a**) Gramine biosynthetic pathway as described in barley. (**b**) Transient expression of *HvAMIS* and *HvNMT* in FAG-95, FAG-98, and FAG-150 leaves. Extracted ion chromatogram for M130 representing the in-source fragment [M−NH_3_+H]^+^ of AMI and gramine is shown. (**c**) Metabolite levels in the leaves of *Fagopyrum esculentum* (accession FAG-95) and *Fagopyrum tataricum* (accession FAG-98, FAG-150) transiently expressing *HvAMIS* and *HvNMT*. Control plants (C) are untransformed wild-type plants. Data are shown as violin plots for C-FAG-95 (N = 14), C-FAG-98 (N = 13), C-FAG-150 (N = 13), transformed FAG-95 (N = 43), FAG-98 (N = 16), and FAG-150 (N = 13). Boxes and whiskers represent interquartile ranges and minimum and maximum values combined with kernel density plots.

To further validate our newly developed protocol for *Agrobacterium*-mediated transient transformation of buckwheat, we transiently expressed the genes responsible for the production of gramine, an allelopathic alkaloid with a fully elucidated biosynthetic pathway in barley [48], in three buckwheat accessions, FAG-95, FAG-98, and FAG-150. For this purpose, a construct encoding the two biosynthetic genes, *HvAMIS* and *HvNMT*, under the control of the CaMV35S and NOS promoters, respectively, was infiltrated into buckwheat leaves. Wild-type and infiltrated leaf material were tested for the presence of AMI (a precursor of gramine) and gramine using UPLC-Q-TOF-MS (LC-MS). While AMI and gramine were not detected in wild-type plants, the expression of *HvAMIS* and *HvNMT* in infiltrated samples resulted in the production of two compounds with retention times and masses matching those of AMI and gramine. Differences in the abundance of these metabolites were observed, with AMI levels being substantially higher than gramine levels in all tested accessions (Figure 3b,c). In plants transformed with the construct containing both transgenes, FAG-95 produced an average of 554.1 pmol/mg FW AMI and 25.3 pmol/mg FW gramine (81 ng/mg and 4.4 ng/mg, respectively), FAG-98 produced an average of 759.4 pmol/mg AMI and 32.6 pmol/mg gramine (111.1 ng/mg and 5.7 ng/mg, respectively), and FAG-150 produced 628.2 pmol/mg AMI and 30.4 pmol/mg gramine (91.9 ng/mg and 5.3 ng/mg, respectively). Additionally, an extra peak was identified in the chromatograms of all three transformed buckwheat accessions, potentially corresponding to MAMI, the methylated form of AMI, and the direct precursor of gramine.

### 2.3. Stable Genetic Transformation of Common and Tartary Buckwheat

**Figure 4:**
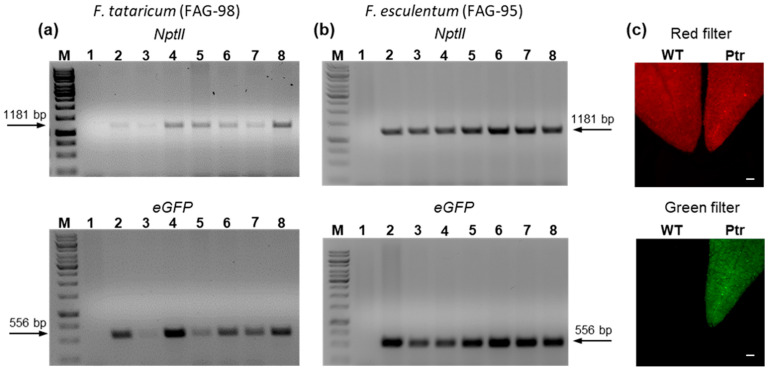
Molecular and fluorescence microscopy analyses of the stable transgenic buckwheat plants expressing the 35SP::GFP_NosP::Kan-pAGM4723 construct. (**a**,**b**) PCR analysis of *F*. *tataricum* and *F*. *esculentum* T1 plants, respectively, using *NptII* and e*GFP* gene-specific primers. Lane M, molecular size marker GeneRuler 1 kb DNA Ladder (Thermo Scientific); Lane 1, non-transgenic wild type plants used as a negative control; Lanes 2–8, the independent transgenic lines. Arrows indicate the amplified *NptII* and *eGFP* fragments, 1181 pb and 556 bp, respectively. (**c**) Fluorescence microscopy images illustrating eGFP expression in the leaf tissues of transgenic Tartary buckwheat plant. The leaves were observed with two different fluorescence filter sets: green and red. Red is autofluorescence of chlorophyll in the leaves of non-transgenic and transgenic buckwheat plants. Green is eGFP fluorescence signal observed in leaf of transgenic plant. WT, wild type FAG-98. Ptr, positive transgenic plant of Tartary buckwheat. The scale bars correspond to 200 µm.

*F*. *esculentum* (FAG-95) and *F*. *tataricum* (FAG-98) were used as recipients for stable transformation using the 35SP::GFP_NosP::Kan-pAGM4723 construct. T1 seedlings were grown on diluted Hoagland medium supplemented with 100 mg/L of Kan (Figure S2d). After two weeks, non-transgenic seedlings showed growth inhibition and began to wilt. The surviving plants were further assessed by PCR amplification of genomic DNA using *NptII*- and *eGFP*-specific primers. True transgenic lines exhibited the expected 1181 bp and 556 bp DNA fragments for the *NptII* and *eGFP* genes, respectively (Figure 4a,b). Leaves of confirmed transgenic lines were further analyzed by fluorescence microscopy, demonstrating eGFP expression in leaf tissues (Figure 4c). Of the 110 T1 seeds of each accession initially analyzed, 19 and 13 plants exhibited transgene integration after PCR screening, resulting in transformation efficiency rates of 17.3% and 11.8% for *F*. *esculentum* (FAG-95) and *F*. *tataricum* (FAG-98), respectively.

## 3. Discussion

Although the genome sequences for common and Tartary buckwheat are now accessible due to sequencing breakthroughs [8,13], progress in understanding gene function is hindered by the lack of an efficient and widely accessible transformation system. Functional gene analysis consequently depends heavily on heterologous expression (e.g., in *Arabidopsis* or *Nicotiana*) [24], but these surrogate systems critically fail to capture buckwheat’s intricate native molecular context, including specific protein–protein interactions and promoter-driven regulation.

Therefore, developing effective methods for direct functional analysis in buckwheat is essential. It is important to note that transformation systems for this species already exist, shifting the focus from whether transformation is possible to how it can be best utilized for research. Our research contributes to refining these techniques for controlled, scientific applications, with the primary goal of employing transformation strictly as a laboratory tool to investigate ecologically and agronomically significant traits—not to promote the commercialization of genetically transformed varieties.

The current study reports simple and effective methods of transient and stable in planta transformation of common and Tartary buckwheat. It was demonstrated that: (1) the *eGFP-GUS* synthetic gene can be successfully expressed in a transient manner in buckwheat cotyledons and leaves using agroinfiltration; (2) the functional recombinant eGFP-GUS protein can be efficiently produced in buckwheat; (3) the novel biosynthetic pathway enabling the production of gramine can be successfully engineered; and (4) stable transgenic plants of Tartary and common buckwheat can be generated using the in planta transformation technique.

Transient transformation is a valuable tool commonly used for functional gene studies, including promoter testing, overexpression or silencing of candidate genes, analysis of subcellular localization, and protein–protein interactions [22]. Moreover, transient expression in plants is widely used for the production of recombinant pharmaceutical proteins [49]. Initially, in the *Fagopyrum* genus, a transient transformation has been developed only for *F*. *esculentum*. Bratić et al. [38] reported transient expression of the *GUS* reporter gene after syringe transformation of leaves. Furthermore, transient expression in common buckwheat leaf and hypocotyl protoplasts was used for functional analysis of buckwheat genes encoding transporters and transcription factors, respectively [23,24,25]. Although the protoplast system provides an opportunity to perform functional analysis of genes of interest in a high-throughput manner, this technique is laborious and requires special skills. In contrast, we developed a relatively simple and efficient transient expression method for *F*. *esculentum* and *F*. *tataricum*. This protocol can be easily performed by an operator with basic training. Previously, syringe agroinfiltration of Tartary buckwheat was successfully used in the study, aiming to reveal the role of *FtMAPK1* in the response to salt stress [42]. When the current manuscript was under review, Wu [43] reported a transient expression method for cotyledons and seeds of Tartary buckwheat, thus allowing analysis of subcellular localization of several proteins encoded by the genes involved in flavonoid metabolism. Moreover, transient overexpression of *FtAGPS1*, a gene involved in starch synthesis in seeds, resulted in an increase in starch content.

*N*. *benthamiana* is a model plant widely used for transient heterologous expression [44]. In most published protocols, the leaves of four- to six-week-old plants of *N*. *benthamiana* plants are usually used for infiltration [50,51]. In our experiments, infiltrated cotyledons of two-week-old plants of common buckwheat demonstrated a very high level of GUS expression (Figure 2). Therefore, the use of buckwheat cotyledons for transient expression assays offers considerable time saving compared to currently employed protocols.

Specialized metabolic pathways in *N*. *benthamiana* can negatively affect its use as a transient transformation system in several ways. One major issue is the potential for metabolic interference, in which the plant’s native pathways interact with the introduced genes, leading to unexpected or reduced expression of the desired proteins. For example, the phenylpropanoid pathway, which is involved in the synthesis of various specialized metabolites, can be altered during agroinfiltration, affecting the stability and accumulation of the target proteins [52]. Additionally, the production of certain metabolites in *N*. *benthamiana* can lead to the formation of by-products, which may interfere with the detection and purification of the desired compounds. This can complicate downstream processing and analysis, making it challenging to obtain pure and functional protein [53]. Furthermore, the plant’s high susceptibility to pathogens can introduce variability in experimental outcomes, as pathogen-induced stress responses can alter metabolic pathways and affect gene expression [54]. When studying plant alkaloid production, *N*. *benthamiana* presents challenges because it naturally produces large amounts of pyridine and piperidine alkaloids, which can interfere with analysis and interpretation [47]. Overall, although *N*. *benthamiana* is a valuable tool for transient gene expression, its specialized metabolic pathways can pose challenges that need to be carefully managed to ensure reliable and consistent results.

The use of buckwheat for the transient expression of genes for metabolic pathway reconstruction is a viable alternative to *N*. *benthamiana*. First, buckwheat has a simpler metabolic profile, which reduces the risk of metabolic interference that can complicate the expression and stability of introduced genes. This could lead to more consistent and reliable results in gene expression studies. Additionally, buckwheat is less susceptible to pathogens than *N*. *benthamiana*, minimizing the risk of pathogen-induced stress responses that could alter the experimental outcomes. Moreover, the large leaves and ease of cultivation make buckwheat suitable for high-throughput experiments and various molecular biology techniques. Its well-characterized genome and the availability of genomic resources further support its use in functional genomics studies. Although buckwheat may not be as widely used as *N*. *benthamiana*, its unique advantages make it a promising alternative for researchers seeking to avoid the drawbacks associated with specialized metabolic pathways in *N*. *benthamiana*, particularly within the new methods developed herein.

In the last decade, significant progress has been made in the synthesis of small molecules via transient expression of non-native genes in plants. Metabolic engineering has traditionally been performed in *N*. *benthamiana*, which has served as a production platform in this field of research [44]. Several reports demonstrated the utility of this platform for production of glucosinolates [55], auxin derivatives [53], alkaloids [56,57], betalains [58], cyanogens [59], and terpenoids [60,61].

Therefore, after the successful transformation of buckwheat using the eGFP-GUS reporter construct, we aimed to test our newly developed protocol to engineer buckwheat to produce heterologous compounds. We took advantage of the recent elucidation of the gramine biosynthetic pathway in barley [48] and transiently expressed *HvAMIS* and *HvNMT* genes under the control of 2x CaMV 35S and Nos promoters, respectively, in the leaves. LC-MC analysis confirmed the de novo production of gramine by the infiltrated leaves (Figure 3). Infiltrated common and Tartary buckwheat leaves accumulated 81–111 ng/mg FW AMI and 4.4–5.7 ng/mg FW gramine. Notably, the amount of AMI was on average 34-fold greater than that of gramine. Possible explanations for this observed difference may be the varied strength of the CaMV 35S and Nos promoters or differences in the catabolic activities of the NMT and AMIS enzymes. Indeed, Sanders [62] reported that the CaMV 35S promoter provided a 30-fold higher accumulation of *NPTII* transcripts than the NOS promoter, and this difference resulted in 110-fold higher NPTII enzyme activity in *Petunia* leaves expressing the CaMV 35S::*NPTII* construct. In summary, our results demonstrate the engineering of gramine production in buckwheat. The developed method allows accelerating the implementation of metabolic engineering in buckwheat. However, the observed imbalance in the accumulation of AMI and gramine can be considered as a drawback of the currently used plant expression cassette. Using the inducible, tissue-specific promoters and promoters containing stress-responsive elements is considered to be a promising approach to control transgene expression [63,64]. We are planning further steps in this direction in order to improve the production of valuable metabolites in buckwheat.

Efficient genetic transformation is crucial for understanding gene function and accelerating the plant breeding process [65]. Among *Fagopyrum* species, the first successful stable genetic transformation of common buckwheat cotyledon explants was reported by Miljuš-Djukić et al. [32]. However, even after three decades, genetic transformation of buckwheat is still far from routine. Only two additional reports describing the successful *A*. *tumefaciens*-mediated in vitro transformation of *F*. *esculentum* [33,34] and one of *F*. *tataricum* [31] have been published. To some extent, this situation can be explained by the fact that traditional in vitro transformation is quite laborious and time-consuming. Moreover, it is species- and genotype-dependent [66]. Additionally, tissue culture-based plant transformation is often associated with somaclonal variations (genetic and epigenetic) [67,68].

The present study was initiated to develop an *Agrobacterium*-mediated in planta transformation method to generate stable transgenic buckwheat plants. Inflorescences were vacuum-infiltrated with *A*. *tumefaciens* harboring the binary 35SP::eGFP_NosP::Kan-pAGM4723 vector. The results suggest that stable transgenic plants of *F*. *esculentum* and *F*. *tataricum* could be obtained with a transformation efficiency rate of 17.3% and 11.8%, respectively. *In planta*, the stable transformation of Tartary buckwheat was achieved for the first time, whereas the transformation of *F*. *esculentum* has been reported previously. Kojima [39,40] described the successful generation of transgenic buckwheat plants after inoculating pricked shoot apical meristems with *A*. *tumefaciens* harboring either *GUS* reporter or *MADS*-box genes. The transformation efficiency of this method was claimed to be quite high (up to 36%). Because such meristematic tissue transformation method was applied only twice to the same indigenous Japanese variety, “Shinano No. 1”, it is hard to tell whether the observed transformation efficiency is to be attributed to this particular genotype or transformation method itself. Recently, the *Agrobacterium*-mediated floral dip method was successfully applied to the generation of transgenic *F*. *esculentum* plants expressing the *GFP* gene [41]. The transformation efficiency reported in that study was up to 12.42%, which is comparable to the rate achieved in our experiments. In summary, here we described a tissue culture-independent in planta transformation method for *F*. *esculentum* and *F*. *tataricum*, which can be easily performed by non-expert operators. The simplicity of the method should allow for its broad application in functional gene analysis and the improvement of agronomic traits in buckwheat.

## 4. Materials and Methods

### 4.1. Chemicals and Reagents

Gramine, hordenine, tryptophan and dopamine hydrochloride were purchased from Sigma-Aldrich (Steinheim, Germany). The 3-aminomethylindole was purchased from Fluorochem (Hadfield, UK), DL-noradrenaline from Fisher Scientific (Schwerte, Germany), and N-methyltryptamine from Carbosynth (Compton, UK). Acetonitrile, methanol, and ultrapure water were purchased from Chemsolute (Renningen, Germany), formic acid from J. T. Baker (Gross Gerau, Germany), and chloroform from Roth (Carl Roth GmbH + Co., Karlsruhe, Germany).

### 4.2. Plant Material

Seeds of *F*. *esculentum* (accession FAG-95, FAG-37) and *F*. *tataricum* (L.) Gaertn. (accession FAG-98, FAG-150) were obtained from the German Federal ex-situ Genebank located at the IPK Gatersleben. Seeds were sown on filter paper moistened with water, and after four days, seedlings (one plant per pot) were transferred to 10 cm diameter pots containing commercial soil substrate. Plants were grown in a greenhouse at 23 °C/18 °C day/night, with 42% air humidity and a 16 h per day photoperiod. The pots were watered twice a day. Accessions FAG-95 and FAG-98 have been selected for further stable transformation experiments, as they produced reasonable amount of seeds and moderate above-ground biomass in our greenhouse conditions.

### 4.3. GUS Staining

β-glucuronidase enzyme activity was detected histochemically according to Jefferson [69]. Briefly, samples were incubated overnight at 37 °C in 100 mM sodium phosphate buffer containing 5-bromo-4-chloro-3-indolyl-beta-D-glucoronide (X-Gluc, Duchefa Biochemie, Haarlem, The Netherlands) as a substrate. After incubation, the samples were washed with 70% ethanol to remove chlorophyll, and the stained plant material was photographed. GUS-stained areas were quantified using the image analysis program ImageJ version 1.54 [70]. Pairwise statistical comparisons between accessions were conducted using a *t*-test at a significance level of 0.05.

### 4.4. Plasmids

The plasmid 35SP::HvP450_NosP::HvNMT-pAGM4673, which encodes aminomethylindole synthase (AMIS) and N-methyltransferase (NMT) proteins, was previously reported [48]. The 35SP::P19 and pXK2FS7 expression vectors have been previously described [71]. Plasmids for stable transformation were generated using Golden Gate modular cloning technology [72,73,74]. *eGFP* was cloned downstream of the cauliflower mosaic virus (CaMV) 35S promoter and upstream of the NOS terminator into the level 1 backbone pICH47732. The plant selectable marker gene, neomycin phosphotransferase (*NptII*), under the control of the NOS promoter, was cloned into the level 1 backbone pICH47772. Finally, level 1 modules were assembled into the binary vector pAGM4723 to obtain 35SP::GFP_NosP::Kan-pAGM4723 (Figure 1). All constructs were introduced into the *Agrobacterium tumefaciens* strain GV2260 by electroporation.

### 4.5. Transient Transformation by Agroinfiltration

*A*. *tumefaciens* strain GV2260 harboring one of the following binary vectors: pXK2FS7, 35S::P450-Nos::NMT-pAGM4673 and p35S::p19 was used to infiltrate leaves of three–four week-old soil-grown buckwheat plants. For each experiment, a single colony was used to inoculate 20 mL of YEB medium containing the appropriate antibiotics. Cultures were incubated at 28 °C for 48 h with continuous shaking at 190 rpm. Two milliliters of such pre-culture were transferred into 500 mL of the same medium, and bacteria were grown until optical density (OD600) reached 1.5–1.8. Afterwards, bacterial cells were collected by centrifugation at 5.000× *g* for 15 min and resuspended in an agroinfiltration medium (AIM) containing 10 mM MgCl_2_, 10 mM MES (pH 5.6) and 0.15 mM acetosyringone. The OD600 of the bacterial suspensions was adjusted to 0.6 and then incubated in the dark for 1–2 h with gentle shaking. Immediately prior to infiltration, the *Agrobacterium* suspension harboring a silencing suppressor construct (p35S::p19) was mixed at a 1:2 ratio with the bacterial suspension that carries one of the two protein expression constructs, pXK2FS7 or 35SP::HvP450_NosP::HvNMT-pAGM4673. Infiltration into the abaxial side of the cotyledons and leaves (top two leaves of five independent plantlets) was performed using a needleless 1 mL syringe. After infiltration, the plants were kept under dim light conditions for 2 days and transferred back to normal growth conditions. The infiltrated leaves/cotyledons were harvested on the fourth day after agroinfiltration and immediately used for histochemical staining or frozen in liquid nitrogen for further (immunoblot or HPLC) analyses.

### 4.6. Stable Transformation by Vacuum Infiltration

*A*. *tumefaciens* GV2260 harboring 35SP::GFP_NosP::Kan-pAGM4723 vector (Figure 1) was grown in YEB medium, as described above. The pelleted bacterial cells were resuspended in infiltration medium containing 4.4 g/L Murashige and Skoog medium containing B5 vitamins (Cat. No. M0231, Duchefa, Netherlands), 5% sucrose, 10 mM MES (pH 5.8), 6-Benzylaminopurin (20 µg/L), 0.15 mM acetosyringone and 0.02% Silwet L-77 to obtain 0.6 OD600. Such a concentration of bacterial suspension was routinely used because we observed that a higher concentration (OD600 = 0.9) was often associated with pronounced leaf necrosis. Buckwheat shoots with inflorescences (4–5-week-old plants) of accessions FAG-95 and FAG-98 were submerged into the *Agrobacterium* suspension in a 3 L glass beaker that was placed inside a vacuum desiccator. Vacuum (−0.8/−1.0 BAR) was applied once for 3 min and then rapidly released. An incubation time of 3 min was applied because, in our preliminary experiments, increasing the time of incubation up to 6 min did not result in the higher number of T1 transgenic seedlings. After infiltration, plants were covered with transparent plastic foil and incubated for a further 48 h under dim light conditions (Figure S2a–c). Afterwards, the foil was removed. Plants were transferred to the greenhouse, and in ten days, vacuum infiltration was repeated once again. Transformed plants were grown to maturity, and T1 seeds were harvested.

### 4.7. Selection and PCR Analysis of Stably Transformed Buckwheat Plants

To select the individual transformants, T1 seeds were germinated on filter paper and three-day-old seedlings were transferred to small rockwool cubes soaked with 1/100 Hoagland nutrient solution containing the antibiotic kanamycin (Kan) at a concentration of 100 mg/L (Figure S2d). Plants were cultivated in a climatized chamber at 45% humidity, 20 °C, and a 14 h light/10 h dark photoperiod with a light intensity of 240 µmol·m^−2^·s^−1^. Seedlings that showed healthy growth after two–three weeks of cultivation in the presence of Kan were further analyzed using PCR to confirm their transgenic nature. The *NptII* gene fragment (1181 bp) was amplified using Fr1a_Kan and Re1_NOS-Term primers. The *eGFP* gene fragment (556 bp) was amplified using Fr1_GFP and Re_GFP primers (Table S1). The cycling conditions were as follows: 95 °C for 3 min, followed by 34 cycles of 95 °C for 30 s, 57 °C for 35 s, and 72 °C for 1 min 11 s, and a final elongation at 72 °C for 10 min. The PCR products were separated on 1% agarose gel. PCR fragments of the expected size were purified using the NucleoSpin Gel and PCR Clean-up Kit (Macherey-Nagel, Düren, Germany), cloned into the pCR8/GW/TOPO cloning vector (Thermo Fisher Scientific, Carlsbad, CA, USA), and Sanger-sequenced. The sequences were analyzed using SnapGene 7.2.1 software available at snapgene.com.

### 4.8. Detection of GFP by Fluorescence Microscopy

Whole leaves of transgenic plants were examined using a fluorescence microscope Olympus BX61 (Olympus, Tokyo, Japan). Photographs were obtained using an ORCA-ER CCD camera (Hamamatsu, Japan).

### 4.9. Protein Extraction and Western Blot Analysis

The infiltrated leaf and cotyledon tissues were harvested on the fourth day after infiltration and ground into fine powder. For protein extraction, 0.2 g of powder was homogenized in 1 mL of lysis buffer containing 100mM Tris-HCl, 18% glycerol, 0.7% 2-mercaptoethanol, 2 mM phenylmethanesulfonyl fluoride (PMSF, Sigma-Aldrich Chemie GmbH, Steinheim, Germany), and 1x protease inhibitor cocktail (PIC, Roche Diagnostics, Mannheim, Germany), pH 6.8. The samples were incubated on ice with shaking for 20 min and centrifuged at 13,000 rpm at 4 °C for 15 min. The protein concentration in the supernatant was determined using the Bradford assay [65]. The crude extracts from each sample (30 μg of protein) were separated on an 8% SDS gel [66] and transferred onto Immobilon-FL PVDF membrane (Merck KGaA, Darmstadt, Germany) by wet electroblotting. Membranes were blocked in TBS (50 mM Tris-HCl pH 7.8, 150 mM NaCl, 1 mM MgCl_2_) containing 5% skimmed milk at room temperature for 1 h, and then probed overnight at 4 °C with a rat monoclonal anti-GFP antibody (1:1000; Chromotek, 3H9). Incubation with IRDye 680RD goat anti-rat secondary antibody (1:1000, LI-COR, 925-68076) was performed at room temperature for 1 h, followed by extensive washing with TBS buffer supplemented with 0.1% Tween-20. The washed membranes were scanned using an Odyssey infrared imaging system (LI-COR Biosciences GmbH, Bad Homburg, Germany).

### 4.10. Buckwheat Leaf Collection for Chemical Analysis

Infiltrated buckwheat leaves, both from the main stem and leaves from side shoots, were collected on the fourth day after agroinfiltration in 2 mL Eppendorf tubes and flash-frozen in liquid nitrogen.

### 4.11. Buckwheat Sample Preparation for Chemical Analysis

Extractions were performed according to the protocol described by Leite Dias [75]. Three steel beads (Ø 3 mm) were added to each tube containing the nitrogen flash-frozen material, and the plant material was ground for 45 s at 30 Hz using a tissue homogenizer (Retch MM400, Retch GmbH, Haan, Germany). Additional liquid nitrogen was added to the samples, and grinding was repeated. The beads were removed and ~50 mg of the frozen samples was weighed. All solvents were obtained from Carl Roth GmbH + Co. KG, Karlsruhe, Germany. All samples were stored at −80 °C until extraction. The extraction phase was initiated by adding 4 µL of pure LCMS-grade methanol/mg fresh weight per sample. Eppendorf tubes were vortexed and incubated at 4 °C overnight. The following day, the samples were centrifuged at 15,000 rpm and 4 °C for 10 min. The supernatant was transferred to new tubes, and the remaining leaf tissue was resuspended in LCMS-grade methanol and incubated at 4 °C for 2–3 h. Afterward, the centrifugation was repeated, and the supernatant was transferred, which was added to the one previously isolated. The extracts were stored at −20 °C until analysis. Before injection, all samples were centrifuged again at 15,000 rpm and 4 °C for 10 min and transferred into UPLC vials.

### 4.12. Instrumentation and Conditions

The samples were analyzed by RP-UPLC (reversed-phase ultra-performance liquid chromatography) ESI-UHR-QTOF-MS (electrospray ionization-ultra-high-resolution-quadrupole time-of-flight mass spectrometry) on an Acquity UPLC system (Waters Corporation, Milford, MA, USA) coupled to an Acquity Fluorescence Detector and a maXis Impact ESI-QTOF MS (Bruker Daltonik GmbH, Bremen, Germany). The system has a 740001685-TAP Acquity solvent manager (1 PM) and a sample manager (740001698-TAP (1 PM)). Sample injection was performed using a PLNO (Partial Loop with Needle Overfill) with an injection volume of 5 µL. The separation was achieved on a Waters Acquity UPLC HSS T3 (2.1 mm × 100 mm, 1.8 µm) column coupled to Acquity UPLC HSS T3 VanGuard (1.8 µm, 2.1 × 5 mm; Waters, Germany) pre-column, at 30 °C and a flow rate of 0.4 mL min^−1^. The initial mobile phase consisted of eluents A (0.1% *v/v* formic acid in water) and B (0.1% *v/v* formic acid in acetonitrile). The total analysis run time was 15 min/sample, where elution started with 99.9% A. From the third minute, this concentration dropped to 95% in favor of acetonitrile containing 0.1% formic acid (B). From the sixth to the tenth minute, the A ratio was 87.4 versus the 12.6 of solution B; consequently, the elution flow was maintained at 50–50 for 30 s. The last 4.5 min were used for washing and re-equilibration of the instrument for the following injection: the elution gradient was composed of 1% A and 99% B for two minutes and was later brought to 99.9% A and 0.1% B.

Detection was performed using a 740002848-TAP Acquity Fluorescence Detector (1 PM) set at an excitation wavelength of 280 nm, an emission wavelength of 320 nm with a PMT gain of 1, and a data rate of 1. The mass spectrometric detector was run in MS1 mode with an electrospray ionization interface in the positive mode. The gas used for desolvation was nitrogen, with the nebulizer pressure set at 8 bar. The dry gas flow rate was set at 8 L/min, and the temperature was maintained at 200 °C. Capillary voltage was set at 4000 V and target mass at 50–1000 *m*/*z*. LC-FLD-MS data were acquired using Compass HyStar 3.2 SR2 software and manually inspected using the Compass DataAnalysis 4.4 SR1 package (Bruker Daltonik GmbH). Extracted ion chromatograms were obtained, which corresponded to the [M+H]^+^ of each compound of interest. The peak area for each compound was curated manually, and the values were quantified using QuantAnalysis 4.4 software (Bruker Daltonik GmbH, DE). In the case of gramine and AMI, the main fragment was not the protonated molecule but an in-source fragment with an m/z of 130.0666. Extracted ion chromatograms for this ion were also used to determine quantity.

### 4.13. Standards and Calibration Curves

A total of 10 mM stock solutions of gramine (GRA, 1.74 mg/mL), hordenine (HOR, 1.65 mg/mL), 3-Aminomethylindole (AMI, 1.46 mg/mL), tyramine (TYR, 1.37 mg/mL), N-methyltryptamine (MeTRY, 1.74 mg/mL) were prepared in methanol/water (80:20). Solutions were further diluted in 0.1% formic acid to create 1 mM solutions. The 10 µM working standard solution was prepared by dilution as a mixture of HOR, TYR, AMI, GRA, and Met-Try in 0.1% formic acid. This standard mix was stored in 200 µL aliquots at −20 °C. Before use, the mixture was heated to room temperature. Calibration standards were constructed by injections in the range 0.05–10 µM for gramine and AMI (0.05, 0.1, 0.2, 0.5, 10 µM).

## 5. Conclusions

Here, we have established two new methods for (i) transient gene expression and (ii) stable genetic transformation of *Fagopyrum esculentum* and *Fagopyrum tataricum*. The very high efficiency of the described methods, combined with their simplicity, allow the use of buckwheat as a reliable system for gene functional validation without the need for expensive infrastructure or complicated procedures.

Our protocols serve as a valuable tool for buckwheat breeding and the generation of engineered genotypes with higher levels of compounds beneficial for human health, such as rutin or quercetin. Furthermore, we propose buckwheat as a compelling alternative to *Nicotiana benthamiana* as a powerful platform for functional validation of genes and synthetic biology in plants, without the high content of pyridine alkaloids, such as nicotine, that can complicate the downstream purification of the product of interest [76].

## Data Availability

The datasets used and/or analyzed during the current study are available from the corresponding author upon reasonable request.

## Supplementary Materials

The following supporting information can be downloaded at: https://www.mdpi.com/article/10.3390/ijms26094425/s1.
