# Supplementary material for: Efficient Agrobacterium-Mediated Methods for Transient and Stable Transformation in Common and Tartary Buckwheat
Source: Int J Mol Sci. 2025 May 6;26(9):4425. doi: 10.3390/ijms26094425 (PMC12072717; doi:10.3390/ijms26094425)
Supplement: Supplementary file 1 [file ijms-26-04425-s001.zip › ijms-3529075-supplementary.pdf]

International Journal of
Molecular Sciences

MDPI

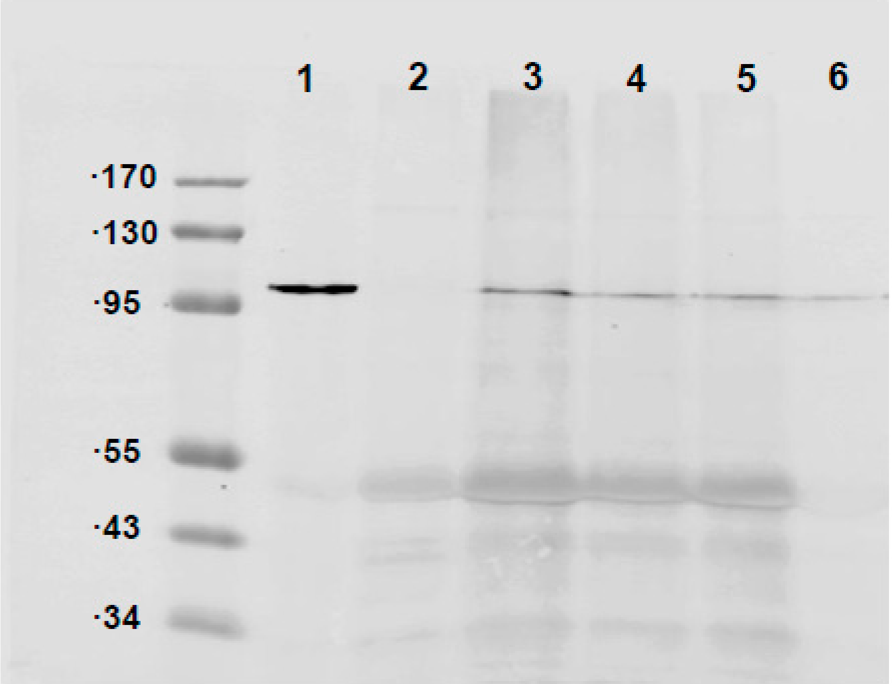

Figure S1. eGFP-GUS protein expression in transfected buckwheat leaves. Plants were infiltrated with *Agrobacterium tumefaciens* GV2260 harboring pXK2FS7 construct. Extracted total soluble proteins were analyzed by Western blotting using anti-GFP antibody. Lane 1, positive control, infiltrated leaf tissue of *Nicotiana benthamiana*; Lane 2, negative. control, non-infiltrated leaf tissue of common buckwheat; Lanes 3-4 and Lanes 5-6, infiltrated leaf tissues of *Fagopyrum esculentum* (FAG-95) and *Fagopyrum tataricum* (FAG-98), respectively. Numbers on the left indicate molecular size markers (kDa).

Table S1. List of the primers used to prove the integration of T-DNA.

Name	Sequence 5'→3'
Fr1a_Kan	AGCTGTGCTCGACGTTGTCACTGAA
Re1_NOS Term	GCGCGCGATAATTTATCCTAGTTTGCG
Fr1_GFP	TGGTGAGCAAGGGCGAGGAG
Re_GFP	TTCTGCTGGTAGTGGTCGGCG

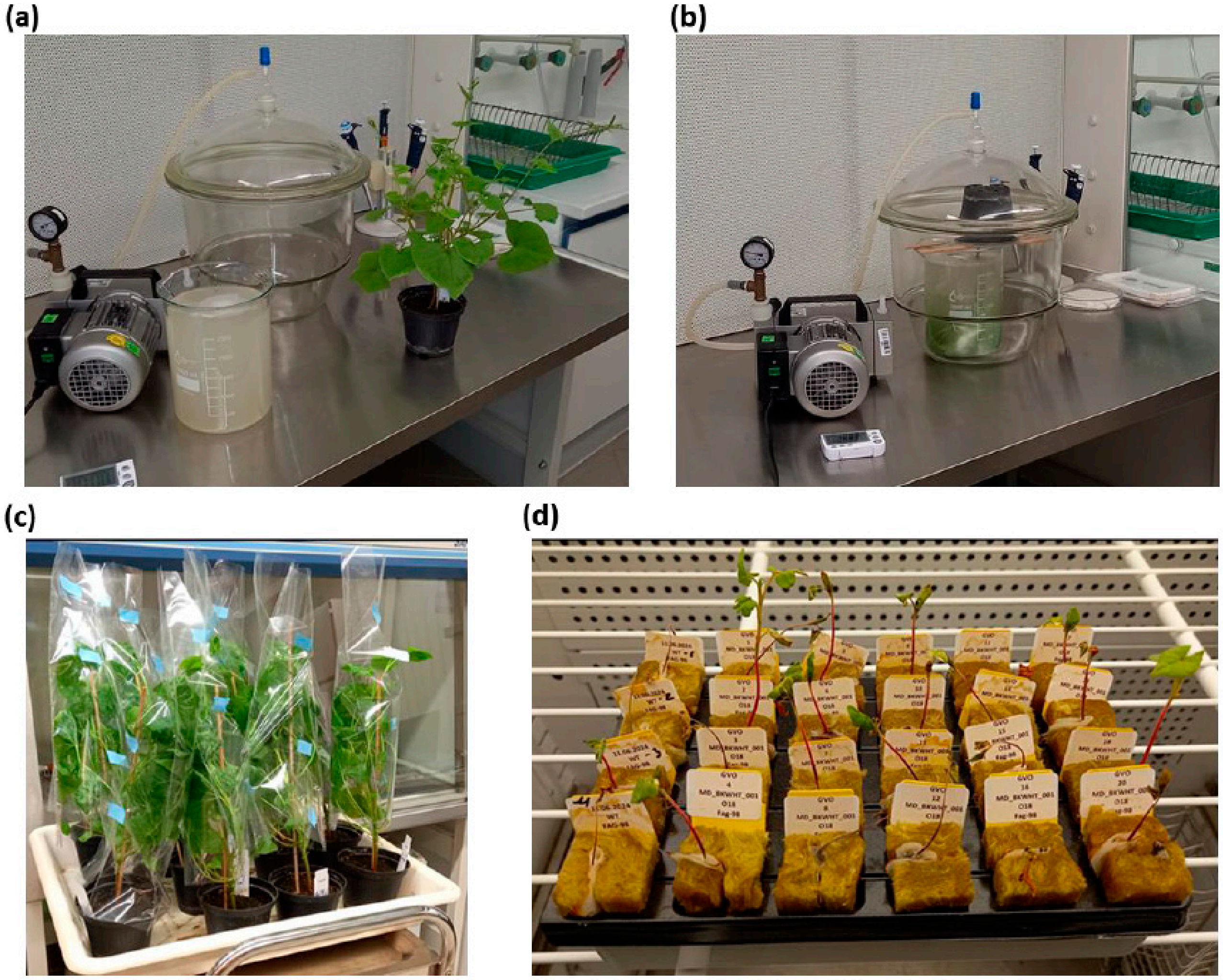

Figure S2. The in planta transformation of buckwheat. (**a**,**b**) *Agrobacterium*-mediated vacuum infiltration of buckwheat. (**c**) Infiltrated plants wrapped with transparent plastic foil. (**d**) Selection of T1 seedlings on diluted Hoagland medium supplemented with 100 mg/L of Kan.
